# Diffusion tensor MRI of the human heart *In Vivo* with a navigator based free breathing approach

**DOI:** 10.1186/1532-429X-14-S1-P238

**Published:** 2012-02-01

**Authors:** Sonia Nielles-Vallespin, Choukri Mekkaoui, Peter D Gatehouse, Timothy G Reese, Jennifer Keegan, Steven Collins, Peter Speier, Thorsten Feiweier, Marcel P Jackowski, David E Sosnovik, David N Firmin

**Affiliations:** 1 CMR Unit, Royal Brompton And Harefield NHS Foundation Trust, London, UK; 2 Martinos Center for Biomedical Imaging, Massachusetts General Hospital, Charlestown, MA, USA; 3 MR R&D, Siemens AG Healthcare Sector, Erlangen, Germany; 4 Institute of Mathematics and Statistics, University of São Paulo, São Paulo, Brazil

## Summary

The purpose of this work was to implement prospective navigators to allow free breathing *in vivo* DTI of the heart to be performed and, thereby, allow the technique to be broadly applied in patients with cardiovascular disease.

## Background

Diffusion tensor MRI (DTI) provides a non-invasive approach for the depiction of the myocardial fibre architecture [1-5]. *In vivo* DTI remains extremely challenging due to the need for motion correction. Several techniques have been used to compensate for respiratory motion: multiple breath-holds (>36 per patient), synchronised breathing and retrospective navigators based on image cross-correlation [2-5]. The purpose of this work was to implement prospective navigators to allow free breathing in vivo DTI of the heart to be performed and, thereby, allow the technique to be broadly applied in patients with cardiovascular disease.

## Methods

The diffusion weighted (DW) STEAM single shot EPI sequence was implemented on a clinical scanner (3T, MAGNETOM Skyra, Siemens AG, Germany) [7]. The crossed slices prospective navigators were applied before and after the STEAM module. The navigator accept/reject algorithm was modified to prevent bulk respiratory motion artifacts in the diffusion encoded images. A biofeedback mechanism was implemented to increase scanning efficiency. Eight volunteers were scanned with breath-hold (BH) and navigated free breathing (FB) protocols (6 diffusion encoding directions, b=350s/mm^2^, TR/TE=1100/23ms, BW=2442Hz/pixel, spatial resolution=2.7x2.7x8mm^3^, 3 slices, 8-10 averages). FA and MD were calculated for 4 sections of the left ventricle (LV) and one of the right ventricle (RV). MD and FA values acquired with the BH and FB techniques were compared with a paired t-test.

## Results

**Figure 1:**
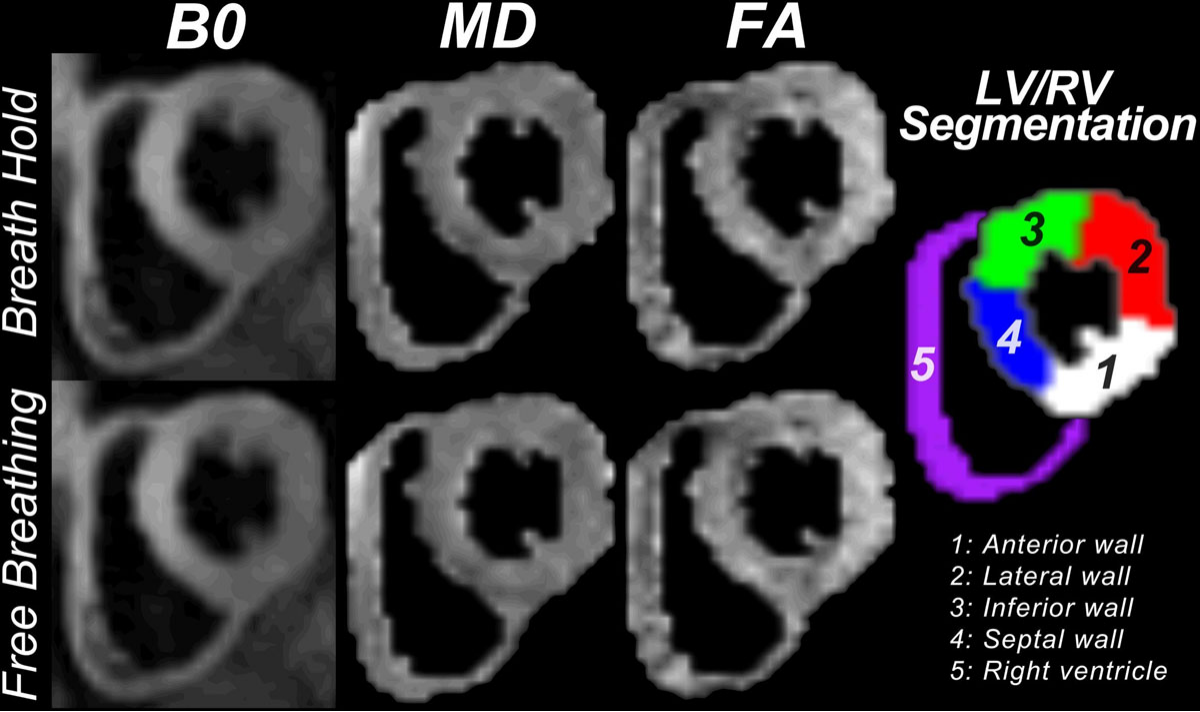
Example b0 image (averaged), FA and MD maps for BH and FB.

**Figure 2:**
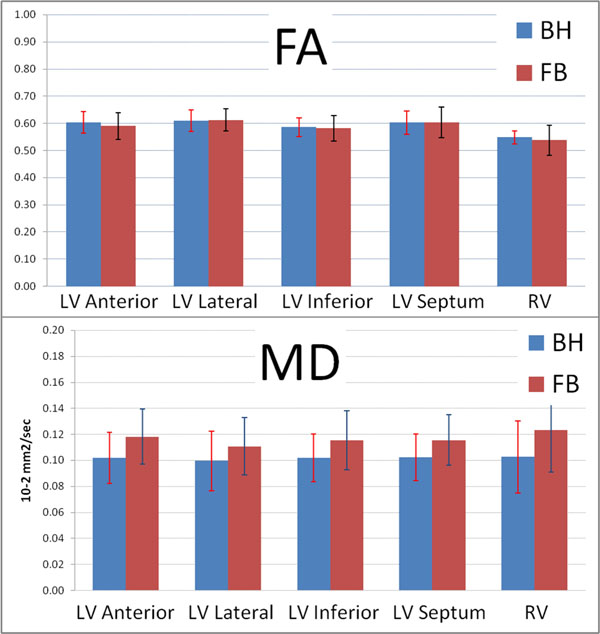
Mean ± SD of FA and MD values for the different regions of the heart.

Scan duration with the BH approach was 14.4±1.5min and 17.1±4.2 min with the FB approach. The FB approach thus significantly improved volunteer comfort without a major increase in scan duration. An averaged b0 image and FA and MD maps are shown in Figure 1. Figure 2 shows the mean±SD of FA and MD values for the different regions of the heart. No significant differences are seen between the BH and FB techniques (p>0.3).

## Conclusions

We show here that a free-breathing navigator based approach to DTI produces high quality in vivo images. The ability to perform free breathing DTI will be useful in normal volunteers but critical if the use of DTI is to be extended to patients with cardiovascular disease and limited breath-hold capacity.

## Funding

This project was funded and supported by the NIHR Biomedical Research Unit at the Royal Brompton and Harefield NHS Foundation Trust and Imperial College London, and by the following grant from the National Institutes of Health (R01HL093038).
